# Non-viral immune electrogene therapy induces potent antitumour responses and has a curative effect in murine colon adenocarcinoma and melanoma cancer models

**DOI:** 10.1038/gt.2014.95

**Published:** 2014-11-06

**Authors:** P F Forde, L J Hall, M de Kruijf, M G Bourke, T Doddy, M Sadadcharam, D M Soden

**Affiliations:** 1 Cork Cancer Research Centre, Leslie C Quick Laboratory, BioSciences Institute, University College Cork, Cork, Ireland; 2 Norwich Medical School, University of East Anglia, Norwich, UK

## Abstract

Antitumour efficacy of electroporated pEEV, coding for granulocyte–macrophage colony-stimulating factor and the B7-1 costimulatory immune molecule (pEEVGmCSF-b7.1) in growing solid tumours, was investigated and compared with a standard plasmid. Application of pEEVGmCSF-b7.1 led to complete tumour regression in 66% of CT26-treated tumours and 100% in the B16F10-treated tumours at day 150 post-treatment. pEEVGmCSF-b7.1 treatment was found to significantly enhance levels of both innate and adaptive immune populations in tumour and systemic sites, which corresponded to significantly increased tissue levels of proinflammatory cytokines including interferon-γ (IFN-γ) and interleukin-12 (IL-12). In contrast, pEEVGmCSF-b7.1 treatment significantly reduced the T-regulatory populations and also the anti-inflammatory cytokine IL-10. Upon further characterisation of functional immune responses, we observed a significant increase in cytotoxic (CD107a+) and IFN-γ-producing natural killer cells and also significantly more in IL-12-producing B cells. Importantly, splenocytes isolated from pEEVGmCSF-b7.1-treated ‘cured' mice were tumour-specific and afforded significant protection in a tumour rechallenge model (Winn assay). Our data indicate that electroimmunogene therapy with the non-viral pEEVGmCSF-b7.1 is able to induce potent and durable antitumour immune responses that significantly reduce primary and also secondary tumour growth, and thus represents a solid therapeutic platform for pursuing future clinical trials.

## Introduction

The current standard of care for cancer uses surgery, radiation and chemotherapy to achieve local tumour control and reduce the risk of disease recurrence.^1^ Immunotherapy is potentially a new therapeutic pilar, which can complement the current standard of care and can reduce risk of disease recurrence.^2, 3, 4, 5^

Immunotherapy-based therapies have the potential to activate a tumour antigen-specific response, which can help to eradicate the tumour and reduce the risk of disease recurrence.^6, 7, 8, 9^ Delivering immunotherapies clinically can be achieved through a number of approaches including the use of gene therapy, which has many applications and methodologies already developed for cancer treatment.^10, 11, 12, 13^ For gene therapy to be successful, safe and efficient gene delivery is critical.^12^ In current cancer gene therapy studies, viral vectors are used in the majority of gene delivery approaches, as they have high-efficiency transfection.^14, 15^ However, there are a number of significant drawbacks that include efficiency of production, host immunogenicity, integration and safety.^15, 16^ An alternative option to viral vectors is plasmid DNA. Toxicity is generally very low, and large-scale production is relatively easy.^17^ However, a major obstacle that has prevented the widespread application of plasmid DNA is its relative inefficiency in gene transfection.^17, 18^ Therefore, most applications for plasmid DNA have been limited to vaccine studies, with a few exceptions.^18, 19^ Consequently, methods that can significantly increase plasmid DNA transfection efficiency will greatly extend the utility of this promising mode of gene transfer. The technique of electroporation is widely used *in vitro* to effectively introduce DNA and other molecules into eukaryotic cells and bacteria. Application of short electrical pulses to the target cells permeabilises the cell membrane, thereby facilitating DNA uptake.^20^ A number of studies, preclinical and clinical, have shown highly successful responses with electroporated plasmid DNA encoding immune genes and also chemotherapeutic drugs.^21, 22, 23, 24, 25^ Recently, we have shown that applying electroporation to a range of tissue types using a new electroporation system, EndoVe in a large pig model. This will significantly enhance the application of electrogene therapy.^26^

Several cytokines have demonstrated significant antitumour effects. Among these, granulocyte–macrophage colony-stimulating factor (*GmCSF*) is one of the most potent, specific and long-lasting inducers of antitumour immunity. *GmCSF* can mediate its effect by stimulating the differentiation and activation of dendritic cells (DCs) and macrophages, and by increasing the antigen presentation capability.^27^ For optimal antigen presentation, engagement of the T-cell receptor with an antigen/major histocompatibility complex requires the costimulatory molecule such as B7-1 (CD80) and B7-2 (CD86).^27, 28^ Subsequently, DC and macrophages process and present tumour antigens to T cells, and to both CD4+ and cytotoxic (CD8+) T cells, by augmenting the antitumour response.^28^ As such, GmCSF is particularly effective in generating systemic immunity against a number of poorly immunogenic tumours.^27^

We recently characterised a non-viral vector therapy system: EEV plasmid (pEEV) with a vastly superior expression capacity when compared with a standard control vector.^26^ The purpose of this study was to test the therapeutic potential of the pEEV system. We hypothesised that pEEV has the capability to reach the therapeutic threshold for the treatment of solid tumours. We present here the use of *pEEV* as a gene therapy vector carrying murine *GmCSF* and human b7-1 genes (pEEVGmCSF-b7.1). We used electroporation as a means to facilitate the delivery of the pEEV and assessed the efficacy and immune induction in primary and secondary responses to treatment in murine colon adenocarcinoma and melanoma cancer models.

## Results

### Electroporation of pEEVGmCSF-b7.1 results in long-term inhibition of tumour growth in CT26 murine colorectal and B16F10 metastatic melanoma models

**Figure 1:**
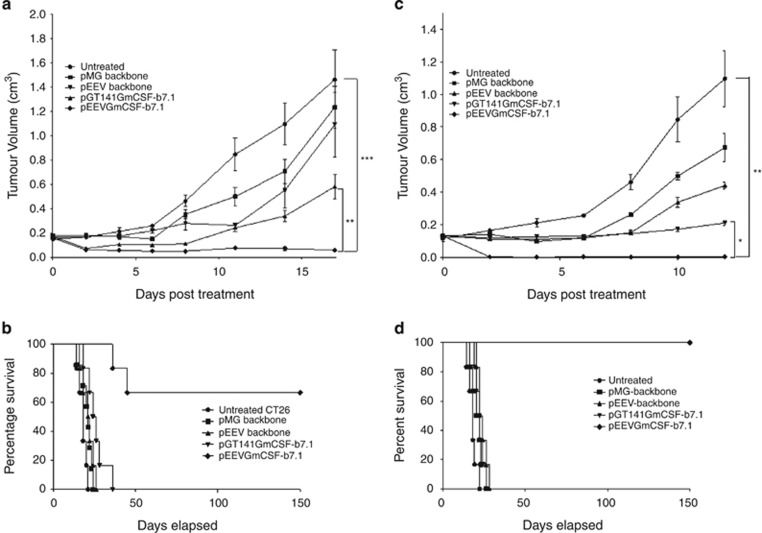
Therapeutic effect on established CT26 and B16F10 solid tumours. (**a**) Representative CT26 tumour growth curve: each Balb/C mouse was subcutaneously injected with 5 × 10^5^ CT26 cells in the flank. On day 14 posttumour inoculation, tumours were treated with pMG (■), pGT141GmCSF-b7.1 (▲), pEEV (▼) and pEEVGmCSF-b7.1 (♦) or untreated (●). Six mice per groups were used and the experiment was performed two times. Tumour volume was calculated using the formula: *V*=*ab*^2^*π*/6. Data are presented as the means±s.e.m. It was observed that the pEEVGmCSF-b7.1 therapy delayed the growth of the tumours most effectively in comparison with the other groups. At 17 days post-treatment, pEEVGmCSF-b7.1 significantly delayed tumour growth compared with untreated tumour (***P<0.0004) standard therapy vector pGT141GmCSF-b7.1 (**P<0.002). (**b**) Representative Kaplan–Meier survival curve of CT26-treated tumours was measured. Only mice treated with pEEVGmCSF-b7.1 survived. Sixty-six per cent of mice survived up to 150 days. All other groups were killed by day 36. (**c**) Representative growth curve of B16F10 tumour. Each C57BL/6J was subcutaneously injected with 2 x10^5^ B16F10 cells in the flank of the mice. On day 15 posttumour inoculation, tumours were treated with pMG (■), pGT141GmCSF-b7.1 (▲), pEEV (▼) and pEEVGmCSF-b7.1 (♦) or untreated (●). Six mice per groups were used and the experiment was performed two times. At 12 days post-treatment, pEEVGmCSF-b7.1 significantly delayed tumour growth compared with untreated tumour (**P<0.0001) standard therapy vector pGT141GmCSF-b7.1 (*P<0.0001). (**d**) Representative Kaplan–Meier survival curve of B16F10 showing pEEVGmCSF-b7.1 had 100% survival up to 150 days post-treatment with all other groups killed by day 28. Similar results were obtained in two independent experiments.

In the current study, we investigated the therapeutic efficacy of pEEVGmCSF-b7.1 when compared with a standard vector also expressing GmCSF-b7.1. To test this, two tumour types (CT26 murine colorectal and B16F10 metastatic melanoma) were treated by electroporating tumours with pMG (standard plasmid backbone), pGT141GmCSF-b7.1 (standard plasmid therapy), pEEV (backbone) and pEEVGmCSF-b7.1 (Figure 1). As expected, the volumes of all non-electroporated (untreated) CT26 tumours and those treated with the empty plasmids, pMG and pEEV, significantly increased (*P*<0.01) in size (Figure 1a). However, we did observe that the empty pEEV plasmid inhibited growth of the CT26 tumour between days 8 and 12, which we have observed previously.^26^ Both therapeutic plasmids delayed the growth rate of the CT26 tumour. Importantly, the growth of pEEVGmCSF-b7.1-treated tumours was significantly more inhibited compared with pGT141GmCSF-b7.1-treated tumours (*P*<0.002) and untreated control tumours (*P*<0.0004). By day 26 post-treatment, the untreated and the pMG- and pEEV-treated groups of animals were euthanised because of tumour size (Figure 1b). Although the standard therapy pGT141GmCSF-b7.1 did inhibit tumour growth, all animals from this group were killed by day 36 when the tumours reached the ethical size of 1.7 cm^3^. One mouse was removed from the pEEVGmCSF-b7.1-treated group on day 36 and again at day 45 because of tumours exceeding the ethical size; however, the remaining 66% of the mice survived up until day 150 post-treatment when they were then killed for subsequent immune analysis. To further test the efficacy of pEEVGmCSF-b7.1 therapy, we used the B16F10 melanoma cell line because of its aggressive nature. Following the same experimental protocol as described for the CT26 model (Figure 1c), we again observed that untreated tumours grew exponentially with the killing of the mice from day 12 onwards (because of tumour size). Again, we observed that pEEVGmCSF-b7.1 treatment delayed tumour growth when compared with the untreated group (*P*<0.0001) and pGT141GmCSF-b7.1 (*P*<0.0001). In terms of survival, the pMG, pEEV, untreated and pGT141GmCSF-b7.1-treated group of animals were killed by day 28 (Figure 1d). Notably, we observed an even greater survival efficacy for the pEEVGmCSF-b7.1 (when compared with the CT26 model) in that 100% mice survived and all remained tumour free for 150 days post-treatment until they were removed for subsequent immune analysis. Similar results were obtained in both tumour types treated based on a range of tumour sizes (Supplementary Figure S2). Taken together, these data indicate that pEEVGmCSF-b7.1 treatment is able to significantly reduce (in the CT26 model) or prevent (B16F10 model) primary tumour growth.

### Treatment with pEEVGmCSF-b7.1 results in robust cellular immune recruitment in systemic and local tumour sites

**Figure 2:**
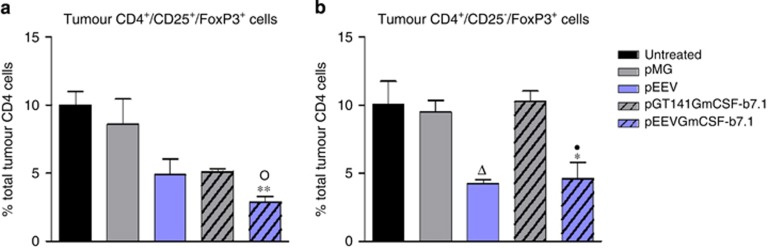
Percentage of the respective T cells found locally at the site of the B16F10 tumours treated with pMG, pEEV, pGT141GmCSF-b7.1 and pEEVGmCSF-b7.1 or untreated. (**a**) Represents data obtained for the CD4^+^CD25^+^FoxP3^+^cells and (**b**) CD4^+^CD25^−^FoxP3^+^ cells. Data represent the mean of the respective cells. Error bars show s.d. from four animals. The asterisks (*) indicate significant values of *P <0.05 and **P <0.01 as determined by one-way analysis of variance (ANOVA) following Bonferroni's multiple comparison of pEEVGmCSF-b7.1 compared with untreated tumour. The asterisks (●) indicate significance values of ^●^*P*<0.05 as determined by one-way ANOVA following Bonferroni's multiple comparison of pEEVGmCSF-b7.1 compared with the standard vector pGT141GmCSF-b7.1. The asterisks (○) indicate significant values of ^○^*P*<0.05 as determined by one-way ANOVA following Bonferroni's multiple comparison of pEEVGmCSF-b7.1 compared with pMG. The asterisks (*) indicate significant values of ^*^P<0.05 as determined by one-way ANOVA following Bonferroni's multiple comparison of untreated compared with pEEV. Similar results were obtained in two independent experiments.

As already indicated, for optimal cancer therapy, robust immune responses must be induced; thus, to determine immune cell recruitment, we performed a comprehensive immune population profile of spleens and tumours 72 h post-treatment. CT26 tumour mice treated with pEEVGmCSF-b7.1 had a significantly greater percentage of splenic CD19^+^ (B cells), DX5^+^/CD3^+^ (natural killer T (NKT) cells), DX5^+^/CD3^−^ (NK cells) and CD8^+^ (cytotoxic T cells) as shown in Table 1. Within the tumour environment, we observed that the percentage of all cell types examined (with the exception of CD4^+^ cells (T cells)) were significantly greater in pEEVGmCSF-b7.1-treated tumours when compared with untreated tumours. Importantly, when the therapeutic plasmid treatments were compared, we observed that pEEVGmCSF-b7.1-treated mice had significantly more splenic and tumour CD19^+^ cells (*P*<0.05) and significantly greater number of tumour DX5^+^/CD3^−^ (*P*<0.001), F4/80^+^ (macrophages) (*P*<0.001) and CD8^+^ (*P*<0.001) cells than control pGT141GmCSF-b7.1-treated mice. We observed a similar immune profile for the B16F10-treated mice (Figure 2b). Splenic and tumour CD19^+^ (*P*<0.001), DX5^+^/CD3^+^ (*P*<0.01), DX5^+^/CD3^−^ (*P*<0.01), CD11c^+^ (DCs) (*P*<0.001), F4/80 (*P*<0.001) and CD8^+^ (*P*<0.001) cells were all significantly higher for the pEEVGmCSF-b7.1-treated mice than for untreated animals. However, we did not observe any significant differences in CD4^+^ or T-cell receptor γδ^+^/CD3^+^ (γδ T cells) (data not shown). Notably, when the standard pGT141GmCSF-b7.1 therapy was compared with pEEVGmCSF-b7.1, the percentage of CD19^+^ (*P*<0.001), DX5^+^/CD3^+^ (*P*<0.01), CD11c^+^ (*P*<0.001) and CD8^+^ (*P*<0.001) cells were all significantly greater, indicating that pEEVGmCSF-b7.1 recruits a superior immune recruitment locally at the tumour site. The spleen data had a similar trend as the tumour data with the percentage of CD19^+^ (*P*<0.01), DX5^+^/CD3^+^ (*P*<0.001), CD11c^+^ (*P*<0.001), F4/80 (*P*<0.001) and CD8^+^ (*P*<0.001) cells all significantly greater in the pEEVGmCSF-b7.1-treated mice when compared with the standard therapy. These data indicate that treatment with pEEVGmCSF-b7.1 induces robust recruitment of innate and adaptive immune cell populations in both colorectal and metastatic melanoma models.

### pEEVGmCSF-b7.1 treatment dampens down suppressive T-regulatory responses

T-regulatory cells (Tregs) are key to dampening effector cell responses, and therefore represent one of the main obstacles to effective antitumour responses. We therefore decided to analyse local Treg percentages in B16F10 tumours of treated animals (Figure 2). In the pEEVGmCSF-b7.1 treatment group, CD4^+^CD25^+^FoxP3^+^ (Treg) cell percentage was reduced significantly (*P*<0.01) compared with the untreated group (Figure 2a). Interestingly, we also observed a CD4^+^CD25^−^FoxP3^+^ tumour cell population, which was also significantly reduced in the pEEVGmCSF-b7.1-treated animals when compared with untreated tumours (*P*<0.05), and also in the therapeutic control pGT141GmCSF-b7.1. These data show that electroporation with pEEVGmCSF-b7.1 reduces potentially detrimental suppressive tumour Treg populations. Control plasmids also had an effect on Tregs, with the pEEV also having an effect on the CD4^+^CD25^−^FoxP3^+^ compared with the untreated tumour (*P*<0.05).

### Mice receiving pEEVGmCSF-b7.1 treatment have a proinflammatory cytokine milieu in spleens and tumours

**Figure 3:**
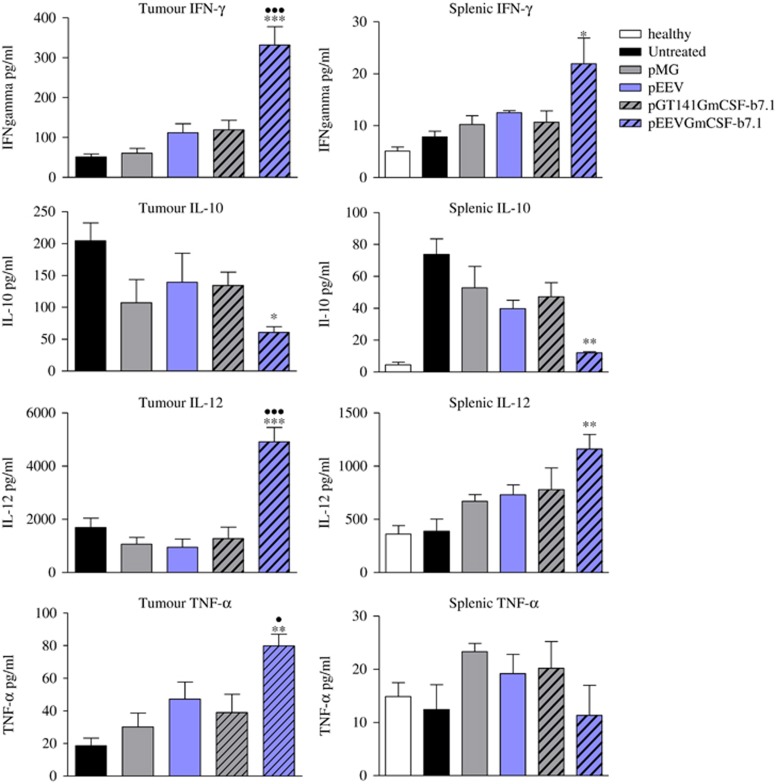
Cytokine levels (IFN-γ, IL-10, IL-12 and Tumour tumour necrosis factor-α (TNF-α)) as measured from tumour and spleens isolated from B16F10 tumour-challenged treated, untreated and healthy mice. The error bars represent the mean of four individual mice±s.e.m. The significance of differences was determined by one-way analysis of variance (ANOVA) following Bonferroni's multiple comparison (*P<0.05, **P<0.01, ***P<0.001 untreated versus pEEVGmCSF-b7.1 and ^●^*P*<0.05, ^●●^*P*<0.01, ^●●●^*P*<0.001 pGT141GmCSF-b7.1 versus pEEVGmCSF-b7.1. Similar results were obtained in two independent experiments.

The cytokine milieu systemically and in the local tumour environment is often indicative of prognosis. Thus, we next examined the concentrations of cytokines within tumours and spleens in treated, untreated and healthy animals 72 h post-treatment (Figure 3). Tumour and spleen concentrations of interferon-γ (IFN-γ) and interleukin-12 (IL-12) were all significantly elevated for the pEEVGmCSF-b7.1 treatments compared with untreated (*P*<0.001), pGT141GmCSF-b7.1 (*P*<0.001) and all other groups analysed. Tumour necrosis factor-α levels were also significantly greater in pEEVGmCSF-b7.1-treated mice when compared with untreated (*P*<0.01), pGT141GmCSF-b7.1 (*P*<0.05) and all other groups analysed. In contrast, IL-10 levels were significantly reduced in pEEVGmCSF-b7.1-treated mice (both in the spleen and tumour) when compared with the pGT141GmCSF-b7.1-treated group (*P*<0.05) and all other groups analysed. Thus, pEEVGmCSF-b7.1 treatment appears to drive a strong inflammatory environment in both systemic and tumour sites, potentially via modulation of immune cell recruitment.

### pEEVGmCSF-b7.1-treated mice have enhanced NK cell and B-cell responses

**Figure 4:**
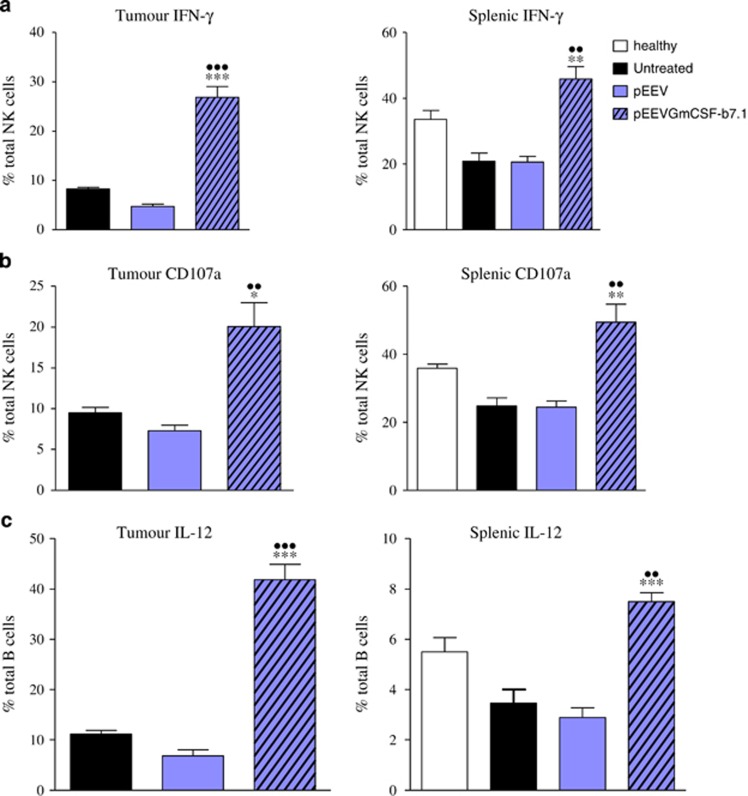
Cytotoxicity of NK and B cells in the tumour and spleens of treated C57BL/6J mice. Data represent the mean of the respective cells. Error bars show s.d. from four animals. The asterisks (*) indicate significant values of *P <0.05, **P <0.01 and ***P <0.001 as determined by one-way analysis of variance (ANOVA) following Bonferroni's multiple comparison pEEVGmCSF-b7.1 compared with untreated tumour. The asterisks (●) indicate significance values of ^●●^*P*<0.01 and ^●●●^*P*<0.001 as determined by one-way ANOVA following Bonferroni's multiple comparison of pEEVGmCSF-b7.1 compared with the untreated groups. Similar results were obtained in two independent experiments.

NK cells have been shown to play critical roles in host immunity to cancer. As we observed a significant increase in this effector population in both systemic and tumour sites (Table 1), we further characterised their response in B16F10-challenged mice. We observed that NK cells positive for IFN-γ were significantly higher (*P*<0.001) in the pEEVGmCSF-b7.1-treated groups when compared with untreated and pEEV control tumours (Figure 4). We observed a similar trend in the splenic NK population with significantly elevated levels of IFN-γ-positive NK cells compared with the untreated and healthy mice. Importantly, when we analysed CD107a (LAMP-1), which is a sensitive marker of NK cell degranulation/cytotoxic activity, we observed significantly higher (*P*<0.001) levels of CD107^+^ NK cells in pEEVGmCSF-b7.1-treated groups when compared with untreated and pEEV control tumours. The role of B cells in antitumour responses has been somewhat overlooked; however, B cells can function as effector cells that mediate tumour immunity and destruction. Interestingly, we observed a significant increase in B-cell percentages both in the spleen and tumour (Table 1), and upon further investigation, we noted that there was also a significant increase in B cells positive for IL-12 in pEEVGmCSF-b7.1-treated groups when compared with controls (Figure 4). These data indicate that pEEVGmCSF-b7.1 promotes the activation and function of NK cells and B cells during B16F10 challenge.

### pEEVGmCSF-b7.1 treatment also promotes antigen-specific secondary tumour protection upon rechallenge

**Figure 5:**
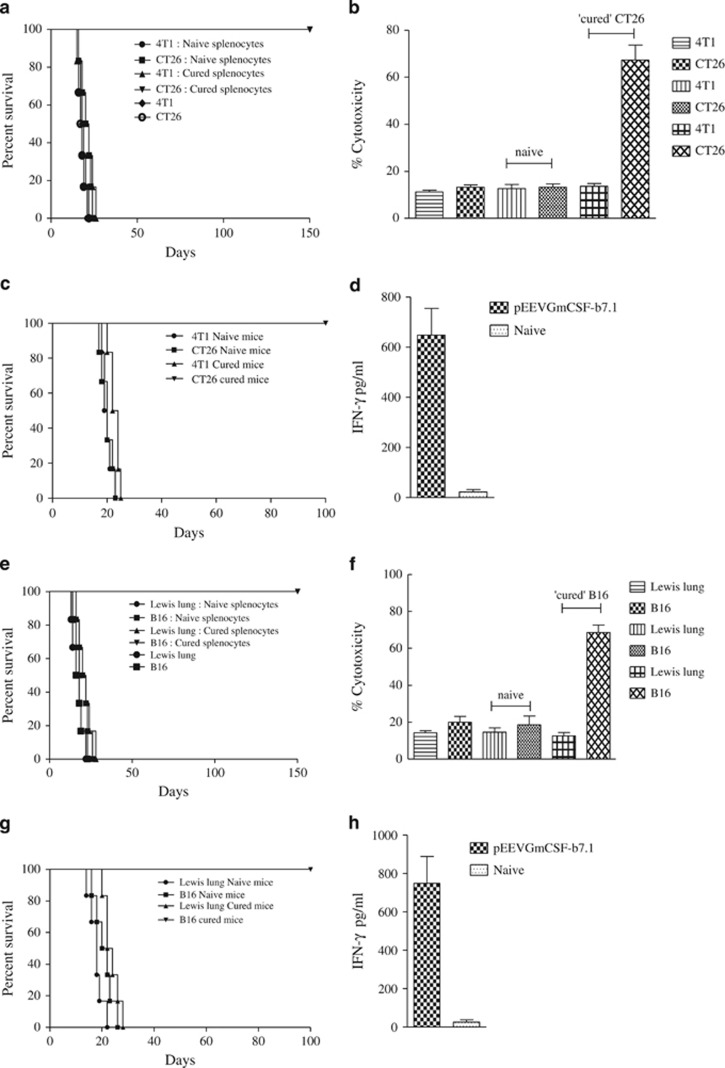
Tumour protection, cytotoxicity and immune memory. (**a**) Tumour protection was observed in the pEEVGmCSF-b7.1-treated CT26 mice when challenged (subcutaneously) with 1 × 10^6^ tumour cells (Balb/C *n*=6 per group) in the left flanks. ‘Cured' and naive mice were challenged with CT26 and 4T1 tumour cells. These mice were monitored for tumour development. One hundred per cent survival was observed in the CT26 cured mice challenged with CT26. All other groups were killed because of tumour burden by day 25. Similar results were obtained in two independent experiments. (**b**) Augmentation of the *in vitro* cytolytic activities of the spleen after pEEVGmCSF-b7.1 treatment of CT26 tumours; the specific cytotoxicity was greatest at an effector:target ratio of 50:1 after 48 h incubation. Groups included CT26, 4T1 cells and naive and ‘CT26 cured' splenocytes incubated with CT26 and 4T1 cells, respectively. The highest cytotoxicity was observed in the CT26 cells incubated with splenocytes obtained from ‘CT26 cure' mice treated with pEEVGmCSF-b7.1. The data shown represent one of two separate experiments with similar results (*n*=6 per group). (**c**) Adoptive transfer of lymphocytes of CT26 study. Mice (*n*=6) received subcutaneous injections of a mixture of mice receiving CT26 cells and splenocytes either from cured or naive mice, a mixture of 4T1 cells and splenocytes either from cured or naive mice and CT26 cells only or 4T1 cells only. All mice receiving mixtures of CT26 cells and splenocytes either from cured or pEEVGmCSF-b7.1 treatment survived up to 150 days, whereas tumours developed in all animals within the other groups. (**d**) Interferon-γ production measured from supernatents obtained from stimulated splenocytes collected from rechallenged—adoptive transfer survivors (50 days post-treatment) and naive animals and IFN-γ was measured. High levels of IFN-γ were produced by pEEVGmCSF-b7.1-treated mice. The y axis represents the concentration of IFN-γ in pg ml^−1^ of the supernatant from the stimulated splenocytes. Error bars show s.d. from six animals. (**e**) Tumour protection was observed in the pEEVGmCSF-b7.1-treated B16F10 mice when challenged (subcutaneously) with 2 × 10^5^ tumour cells (C57BL/6J *n*=6 per group) in the left flanks. ‘Cured' and naive mice were challenged with B16F10 and Lewis lung tumour cells. These mice were observed for tumour development. One hundred per cent survival was observed in the B16F10 cured mice challenged with B16F10. All other groups were killed because of tumour burden by day 28. Similar results were obtained in two independent experiments. (**f**) Augmentation of the *in vitro* cytolytic activities of the spleen after pEEVGmCSF-b7.1 treatment of B16F10 tumours; the specific cytotoxicity was greatest at an effector:target ratio of 50:1 after 48 h incubation. Groups included B16F10, Lewis lung cells and nive and ‘B16F10 cured' splenocytes incubated with B16F10 and Lewis lung cells, respectively. The highest cytotoxicity was observed in the B16F10 cells incubated with splenocytes obtained from ‘B16F10 cure' mice treated with pEEVGmCSF-b7.1. The data shown represent one of two separate experiments with similar results (*n*=6 per group). (**g**) Adoptive transfer of lymphocytes of B16F10 study. Mice (*n*=6) received subcutaneously injections of a mixture of B16F10 cells and splenocytes either from cured or naive mice, a mixture of Lewis lung cells and splenocytes either from cured or naive mice, B16F10 cells only or Lewis lung cells only. All mice receiving mixtures of B16F10 cells and splenocytes either from cured from pEEVGmCSF-b7.1 treatment survived up to 150 days, whereas tumours developed in all animals within the other groups. (**h**) Interferon-γ production measured from supernatants obtained from stimulated splenocytes collected from rechallenged—adoptive transfer survivors (~50 days post-treatment) and naive animals and IFN-γ was measured. High levels of IFN-γ were produced by pEEVGmCSF-b7.1-treated mice. The y-axis represents the concentration of IFN-γ in pg ml^−1^ of the supernatant from the stimulated splenocytes. Error bars show s.d. from six animals.

There is strong evidence that links positive prognosis with robust tumour immune infiltrate in several different cancer types. As we observed a superior immune response after pEEVGmCSF-b7.1 treatment (Figures 2a and b), which was linked to reduced or absent primary tumours, we next challenged these mice (both CT26 and B16F10 cured and naive mice) to determine the overall tumour protection. To compare tumour growth of ‘cured mice', naive age-matched mice were inoculated with the same dose of viable tumour cells (Figures 5a and e). To determine tumour-specific protection, a different tumour was selected and cured, and the naive mice were challenged with either Lewis lung cancer (LLC) or breast cancer (4T1) cells. We observed long-term (100 days) tumour-specific protection in pEEVGmCSF-b7.1-treated ‘cured' mice group both for the CT26 and B16F10 models. Notably, we observed that tumour protection was limited to the CT26 or B16F10 cells and not the previously unexposed tumours such as 4T1 and LLC in the respective models. These data suggest that pEEVGmCSF-b7.1 treatment results in a durable response.

To further confirm an antigen-specific response, we next determined the *in vitro* cytotoxicity of mixed splenic T-lymphocyte population against CT26 and B16F10 cells. (Figures 5b and f). Cytotoxic responses of splenic T lymphocytes were significantly greater against CT26 and B16F10 cells from pEEVGmCSF-b7.1-treated ‘cured' mice than in naive mice. To determine the specificity of this cytotoxicity, we included the unexposed tumours 4T1 and LLC for the respective model. The splenic T lymphocytes against the CT26 and B16F10 demonstrated a low percentage cytotoxicity. These results correspond with the observed immunity *in vivo* (Figures 5a and e).

The possible development of an immune-mediated antitumour activity following pEEVGmCSF-b7.1 was further tested by a modified Winn assay (adoptive transfer), where groups received subcutaneous inoculation of a CT26 or B16F10 cell mixture and splenocytes from pEEVGmCSF-b7.1-treated ‘cured' mice or naive mice, a mixture of 4T1 or Lewis lung cells and splenocytes from pEEVGmCSF-b7.1-treated ‘cured' mice or naive mice, 4T1 or LLC and CT26 or B16F10 in their respective model (Figures 5c and g). All mice inoculated with splenocytes from naive mice developed tumours. Mice inoculated with mixtures of splenocytes and 4T1 or Lewis lung developed tumours, whereas no tumour growth was observed in mice inoculated with splenocytes from pEEVGmCSF-b7.1-treated ‘cured' mice in both the CT26 and B16F10 models, indicating the protective effect was antigen-specific as observed in the *in vitro* analysis. Control groups that were inoculated with CT26, B16F10, 4T1 or LLC cells all developed tumours and indicated that the tumours were growing in the correct manner. The tumour-protective effect in the mice inoculated with splenocytes from pEEVGmCSF-b7.1-treated ‘cured' mice in both the CT26 and B16F10 models resulted in prolonged survival (150 days). We also observed significantly high levels of IFN-γ from animals that received adoptively transferred mixtures of both CT26 and B16F10 cells and splenocytes from the pEEVGmCSF-b7.1-treated ‘cured' mice of the respective model and naive mice of the same age (Figures 5d and h). This suggests adoptive transfer to naive mice of specific antitumour immune response provided protection to tumour challenge.

## Discussion

Cancer treatment strategies using immunotherapy have recently gained clinical traction with the positive results emanating from ipilimumab antibody clinical studies.^29^ The use of DNA plasmid-based gene therapy for immunotherapy has also showed promise with encouraging data reported with IL-12 delivered via electroporation to patients with malignant melanoma.^24^

We have developed a DNA plasmid that enables the enhanced expression of exogenous genes in transfected cancer cells. The pEEV was used to deliver via electroporation DNA encoding for *GmCSF* and *b7.1* (pEEVGmCSF-b7.1), which we have demonstrated protects from primary and secondary tumour growth in both the colon adenocarcinoma and the melanoma cancer models by generating a robust proinflammatory immune cell recruitment and cytokine environment in both systemic and tumour compartments.

Previously, we have shown that delivery of our EEV plasmid (pEEV) via electroporation is capable of achieving reliable and superior expression in a variety of murine and porcine tissue types when compared with a control plasmid.^26^ We therefore decided to engineer pEEV to contain the immune therapeutic genes: *GmCSF* and *b7.1*, and test their efficacy in two aggressive cancer models. As already highlighted, *GmCSF* and *b7.1* are key molecules in inducing robust immune responses, which may facilitate subsequent antitumour immunity.^27, 28^ Indeed, only those mice treated with pEEVGmCSF-b7.1 were observed to have reduced or absent primary tumour growth, which paralleled to significantly improved survival rates. Mice treated with pEEVGmCSF and pEEVb7.1 delivered singly had minimal/no therapeutic effect (data not shown). The control plasmids also had an effect on the tumour growth. Naked plasmid DNA can lead to immune responses. Plasmid sequences containing CpGs can induce strong humoral and cell-mediated immune response. Unmethylated CpG motifs interact with the Toll-like receptor-9 in cells of the innate immune system. This interaction triggers an inflammatory reaction, which, in turn, drives the adaptive response to the vector-encoded protein.^30^ This might explain the slight improvement in tumour volume reduction and survival. The effects of which are short-lived.

Correspondingly, when we analysed immune populations, we observed that pEEVGmCSF-b7.1 treatment induced a significant increase in the levels of innate and adaptive immune cells both systemically and within the tumour environment. More specifically, increases in DC and macrophage levels were observed and may correspond to the presence of high *GmCSF* expression after pEEVGmCSF-b7.1 transfection, as previous studies have shown that GmCSF^−/−^ mice have reduced DC and macrophage recruitment and survival.^31, 32^ Several studies have also indicated that B cells can respond to *GmCSF*.^33^ Within cancer immunology, B cells are currently underinvestigated, but appear to have a complex role. Interestingly, we observed that B-cell populations were increased after pEEVGmCSF-b7.1 treatment, and upon further examination we observed that they had increased expression of IL-12, which correlated with the total tissue levels of IL-12 in the spleen and tumour tissues of treated mice. IL-12 is a potent proinflammatory and antiangiogenic cytokine capable of activating multiple aspects of innate and adaptive antitumour immunity, particularly via modulation of IFN-γ.^34^ Indeed, we also observed increased tissue IFN-γ levels after pEEVGmCSF-b7.1 treatment, which potentially corresponds with the high levels of IFN-γ+ NK cells in pEEVGmCSF-b7.1-treated mice. Importantly, IL-12 and, indeed, *GmCSF* are also able to regulate NK cell-mediated cytotoxicity. NK cells are critical innate cells during cancer as they able to distinguish and destroy malignant cells from healthy cells, which is controlled by complex interactions of inhibitory and activating receptors, which trigger specialised downstream effector signalling pathways.^35, 36^ Importantly, we also observed a significant increase in NK cell levels, and, in tandem, a high proportion of cells expressing CD107a (cytotoxicity marker) in those mice treated with pEEVGmCSF-b7.1, highlighting the potential for pEEVGmCSF-b7.1 treatment to induce IL-12-expressing B cells, which in turn potentially induces potent NK cell tumour killing and NK cell-derived IFN-γ production. Thus, these data emphasise the importance of the presence of these cell populations for cancer treatment and prognosis.

An overall reduction in tumour-resident Tregs is indicated as a positive response for any cancer therapy.^6^ Tregs cells are important regulators of immune cells and are seen as immune suppressors.^6, 27^ Thus, any reduction of Tregs has the potential to allow immune cell recruitment to the tumour site. pEEVGmCSF-b7.1 treatment correlates with Treg reduction with corresponding improved cellular infiltration. Interestingly, we also observed a CD4^+^CD25^−^FoxP3^+^ population that was significantly reduced after pEEVGmCSF-b7.1 treatment. Previous studies have suggested that this population may act as a reservoir/precursor of CD4^+^CD25^+^FoxP3^+^ Tregs; thus, this reduction may assist in the reduced levels of Tregs present in our treated tumours.^27^ Importantly, Treg cells also contribute to the production of immunoregulatory cytokines, such as IL-10.^37^ IL-10 is an immune-suppressing anti-inflammatory cytokine and is upregulated in many cancer models. Additionally, IL-10 downregulates the expression of T_H_1 cytokines, such as IL-12 and IFN-γ, and induces a Treg response.^37, 38^ This reduced level of IL-10 observed in the pEEVGmCSF-b7.1-treated group correlates with the reduced levels of Treg cells and supports the positive inflammatory responses of this therapy. Both pEEV and pGT141GmCSF-b7.1 reduced the levels of Tregs and had no real effect on the cytokines or immune cells. This may be caused because a minimal threshold of GmCSF-b7.1 is needed to be effective and the high expression levels from the pEEVGmCSF-b7.1 is necessary for this therapy to be effective and the degree of expression could be important.

The goal of all anticancer therapies is long-lasting responses that also prevent metastases and secondary tumours. As we observed such potent cellular and cytokine responses after pEEVGmCSF-b7.1 treatment, we studied outcomes after tumour rechallenge. Notably, all naive mice succumbed to disease, whereas those in the pEEVGmCSF-b7.1 group developed no tumours, suggesting protective and long-lasting immunity. As already discussed, *GMCSF* is known to mature both DC and macrophages for antigen presentation. During our studies, an antigen-specific immune response was indeed suggested, as tumour protection and *in vitro* killing was limited to the CT26 or B16F10 and not to the previously unexposed tumours such as 4T1 and LLC in the respective models. Furthermore, DCs and macrophages require help from NK cells for maturation, proper antigen presentation function and priming of T-cell responses.^39^ This bidirectional crosstalk during the early phases of tumour immunity as indicated by our data may also influence the following type, and magnitude of adaptive immune response to pEEVGmCSF-b7.1. B cells are also important in antigen presentation to T-cell populations. Indeed, previous studies have indicated that activated B cells can be used as effective APCs for T-cell sensitisation to tumour antigens.^40^ IL-10 has been shown to affect directly the function of antigen-presenting cells by inhibiting the expression of major histocompatibility complex and costimulatory molecules,^41, 42^ which in turn induces immune suppression or tolerance;^43^ thus; reduced levels into pEEVGmCSF-b7.1-treated mice may also correspond to robust antigen-specific immunity.

Although we did not investigate this mechanism of rechallenge protection in great detail (using mixed lymphocyte preps), we postulate that the antitumour-generated immunity could be because of the action of a few different cell types. The protection could be antigen-specific B cells producing cytokines that activate NK cell and T-cell killing, and, indeed, a previous study has indicated that B cells may mediate tumour regression/protection after adoptive immunotherapy of solid tumours.^44^ Finally, antigen-specific T-cell responses, which are well known to be absolutely crucial for comprehensive anticancer immunity and eradication of tumours, would be a highly likely protective immune mechanism.^6^ Clinically, this protective nature is a very important observation. Recurring tumours post-treatment is a major issue that exists with standard treatments similar to surgery with tumour cells left behind in postoperative margins. This observation again supports the importance of this treatment and as its potential clinical adaption.

Importantly, the electrogene therapy with pEEVGmCSF-b7.1 protocol appears to be safe and non-toxic. All mice remained healthy throughout the course of the experiments and there were no treatment-related deaths. This suggests that electroporation of the solid tumours is well tolerated and the transgene expression did not induce systemic toxicity. Also, it was observed that long-term survival of mice had no evidence of autoimmune disease, suggesting that, in this model, immune clearance of tumours could be achieved while maintaining autoimmune control.

Our findings suggest that the strategy of using pEEV as a therapeutic plasmid coding for two immunogenes, *GmCSF* and *b7.1*, in combination with electroporation compared with plasmids using the cytomegalovirus promoter was far superior. The treatment established potent, durable tumour immunity and had a curative effect in two tumour models tested. As indicated above, a robust immune response is important in cancer treatment outcome and prognosis. We demonstrated that pEEVGmCSF-b7.1 did induce a strong immune response, including enhanced B- and NK cell responses. We can conclude that immunogene therapy of solid tumours by pEEV in combination of electroporation results in containment of the tumour. This strategy could be developed for clinical application.

## Materials and methods

### Cell tissue culture

Tumour cell lines B16F10, CT26, LLC and 4T1 were obtained from the American Type Cell Collection (Manassas, VA, USA). The murine colon adenocarcinoma, CT26 and Lewis lung carcinoma, LLC cell line was cultured with Dulbecco's modified Eagle's media (Sigma, Wicklow, Ireland) supplemented with 10% (v v^−1^) foetal calf serum, 300 μg ml^−1^ L-glutamine. The murine breast carcinoma, 4T1 cell line and the mouse melanoma cell line was cultured in RPMI-1640 (Sigma) supplemented with 10% (v v^−1^) foetal calf serum and 300 μg ml^−1^ L-glutamine. Cells were maintained in a logarithmic phase of growth at 37 °C in a humidified atmosphere supplemented with 5% CO_2_.

### Animals and tumour induction

Female Balb/c and C57BL/6J (6–8 weeks) were obtained from Harlan Laboratories (Oxfordshire, UK). For routine tumour induction, 5 × 10^5^ tumour cells suspended in 200 μl of serum-free Dulbecco's modified Eagle's medium were injected subcutaneously into the flank of the female C57BL/6J and Balb/C mice. Following tumour establishment, tumours were allowed to grow and develop and were monitored by alternate day measurements in two dimensions using vernier callipers. Tumour volume was calculated according to the formula: *V*=*ab*^2^*π*/6, where *a* is the longest diameter of the tumour and *b* is the longest diameter perpendicular to diameter *a*. From these volumes, tumour growth curves were constructed. The mice were killed when the longest diameter of the tumour reach 1.7 cm. Measurement of tumour growth was halted once the first mouse reached the ethically approved size and ethically killed. Survival curves were constructed as a measure of tumour regrowth and survival of the animal.

### Ethics statement

All murine husbandry and experimental procedures were approved by the University College Cork Animal Experimentation Ethics Committee and carried out under licenses issued by the Department of Health, Ireland as directed by the Cruelty to Animals Act Ireland and EU Statutory Instructions.

### Plasmids

The pMG plasmid was purchased from InvivoGen (Toulouse, France). A version of this plasmid, designated pGT141, containing the murine *GMCSF* and human *B7-1* genes transcriptionally controlled from the hEF1-HTLV and cytomegalovirus promoters, respectively, was designed and cloning was performed on contract by InvivoGen. The EEV plasmid was created by incorporating a Semliki Forest virus DNA replicase sequence (kindly donated by Prof Greg Atkins, Virus Group, Department of Microbiology, School of Genetics and Microbiology, Trinity College, Dublin, Ireland). A nuclear localisation sequence was also incorporated to allow for nuclear targeting.^26^ The murine *GMCSF* and human *b7.1* genes were also incorporated into the pEEV. Plasmids were propagated in *Escherichia coli* strain Top10 and purified on endotoxin-free Qiagen-tip 500 columns (Qiagen) All plasmids are described in Supplementary Figure S2.

### DNA transfection and *in vivo* electroporation

Electroporation procedure was carried out under general anaesthesia by intraperitoneal administration (100 μl) of 200 μg xylazine and 2 mg ketamine. Fifty micrograms of plasmid DNA in 50 μl sterile injectable phosphate-buffered saline was injected into the tumour. Five minutes after the plasmid injection, the tumour was electroporated using a custom-built 5 needle circular array electrode (Cork Cancer Research Centre, Cork, Ireland). Previously used *in vivo* electroporation parameters were 1200 V cm^−1^, 100 μs pulse length; 1 pulse and 120 V cm^−1^, 20 ms; and 8 pulses at 1 Hz, and were administered in sequence using the *E.Pore Gx* (Cork Cancer Research Centre) square-waved pulse generator.^45^ The high voltage pulse was used to induce electroporation in the cell membrane and the ensuing small voltage pulses were used to create an electrophoretic field to assist movement of the negative charged DNA plasmid across the cells.

### Flow cytomery analysis

Single-cell suspensions from spleens and tumours of individual mice were prepared as described previously.^46^ Cells were added at a concentration of 2 × 10^5^ cells per well (96-well plates) in blocking buffer (1 × phosphate-buffered saline/1% bovine serum albumin/0.05% sodium azide/1% rat, hamster and mouse serum). To this, 50 μl of each monoclonal antibody (mAb) dye mix was added plus 5 μl of amine-reactive viability UV dye (Invitrogen, Toulouse, France) to determine dead cells, with incubation in the dark at 4 °C for 30 min.^47^ The mAb used for flow cytometry are listed in Supplementary Table S1. Cells were washed and resuspended in 200 μl of 3% formalin. To perform flow cytometric analyses, a FACSLSRII 5 Laser (UV/Violet/Blue/Yellow-Green/Red) cytometer and BD Diva software (Becton Dickinson, Dublin, Ireland) were used. For each sample, 50 000-200 000 events were recorded. Background staining was controlled by labelled isotype controls and fluorescence minus one. The results represent the percentage of positively stained cells in the total cell population exceeding the background staining signal. For determination of intracellular cytokine production by leucocytes, cells were incubated for 6 h at 37 °C with BD Activation Cocktail plus GolgiPlug (PMA, ionomycin and brefeldin A (BD Biosciences, Oxford, UK)); unstimulated controls were also set up for each cytokine study. Cells were then washed with staining buffer and stained at 4 °C for 30 min with appropriate surface mAb. Controls were stained with appropriate isotype-matched control mAbs. Cells were then fixed and saponin-permeabilised (Perm/fix solution; BD Biosciences) and incubated with mAb listed in Supplementary Table S1 or isotype-matched control mAbs. After 30 min, cells were washed two times in permealisation buffer (BD Biosciences) and then analysed by flow cytometry, as described above. NK cells were identified as DX5^+^/CD3^−^, neutrophils as Ly6G^+^, DC as CD11c^+^, macrophages as F4/80^+^, B cells as CD19^+^ and T cells as CD4^+^ and cytotoxic T cells as CD8^+^.

### Analysis of cytokine levels

Tumour and spleen homogenates were analysed using mouse proinflammatory 7-plex (IFN-γ, IL-1β, IL-6, IL-10, IL-12p70, KC/GRO/CINC, tumour necrosis factor-α Meso Scale Discovery, Rockville, MD, USA). All assays were performed as per the manufacturer's instructions. Cytokine levels are expressed as pg cytokine per ml (sensitivities of assays >0.5–11 pg ml^−1^).

### Evaluation of antigen specificity and long-term tumour protection

To test recall antitumour responses, naive and cured mice (primary B16F10 or CT26 tumour long term; >100 days) were rechallenged with the same tumourogenic dose of B16F10 or CT26 in the opposite flank and survival monitored over 100 days. To assess the specificity of pEEVGmCSF-b7.1, the treatment was restricted to B16F10 or CT26 cells mice bearing two different tumour types, and Lewis lung carcinoma and 4T1 were also assessed.

### *In vitro* augmentation of cytotoxicity activity

To assay cytotoxicity activity against CT26 and B16F10 tumour cells, mixed splenocytes were harvested from mice responding to GmCSF-B7.1 treatment and resistant to tumour growth on rechallenge with B16F10 or CT26. The spleen was harvested and tumour-specific lymphocytes were induced by incubating, 2 × 10^6^ splenocytes with 2 × 10^5^ mitomycin C-treated tumour cells in the presence of 25 IU ml ^−1^ rmIL2 (Sigma) for 5 days. Lymphoid cells were then harvested, washed three times in serum-free medium and applied as effectors at various effector:target ratios (100:1, 50:1 and 1:1) with 2 × 10^4^ target cells. The 50:1 ratio had optimum effects and therefore data are shown. Cells were incubated overnight in 96-well plates to allow target cell killing. Wells were then washed five times with phosphate-buffered saline to eliminate non-adherent cells (dead and all effector cells). The MTT (3-(4,5-dimethylthiazol-2-yl)-2,5-diphenyl-tetrazolium bromide) assay was used to quantify the emaining living cells. MTT working solution (0.45 mg ml^−1^) was added to each well and cultures were incubated for 4 h at 37 °C. The culture medium was then removed from each well and the precipitates were dissolved with 150 ml of dimethyl sulphoxide (Sigma) for 10 min. Absorbance was read at 570 nm and the percentage cytotoxicity was calculated.

### Statistical analysis

Experimental results were plotted and analysed for significance with Prism 4 software (GraphPad Software Inc., La Jolla, CA, USA). *P*<0.05 was considered significant.

## Figures and Tables

**Table 1:** Percentage of immune cells in all tumour and spleen cells present 72 h post-treatment

*Cell type*	*Tumour*	*Tissue*	*Untreated*	*pMG*	*pEEV*	*pGT141GmCSF-b7.1*	*pEEVGmCSF-b7.1*
CD19^+^	CT26	Tumour	4.0±0.6	5.3±0.6	5.3±0.9	6±0.9	12.7±1.2***/ ^●●^
		Spleen	19.7±2.5	25.6±1.5	23±3.3	36.3±2.4	46.0±2.4***/ ^●^
DX5^+^/CD3^+^		Tumour	2.2±0.1	2.4±0.3	2.2±0.2	3.0±0.6	6.4±0.9**/ ^●●^
		Spleen	0.3±0.1	0.3±0.1	0.2±0.1	0.2±0.1	0.5±0.1*/ ^●●^
DX5^+^/CD3^-^		Tumour	13.0±1.0	10.7±0.9	13.2±0.6	11.8±1.0	22.0±2.0**/ ^●●●^
		Spleen	2.5±0.2	2.9±0.4	2.9±0.3	4.3±0.6	6.1±0.4
CD11C^+^		Tumour	19.7±0.7	23.3±2.1	28.7±2.7	30.4±3.1	41.2±2.3***
		Spleen	10.2±0.2	9.6±0.4	9.9±1.1	9.1±1.5	9.4±1.3
F4/80^+^		Tumour	13.1±0.7	10.7±0.5	12.4±1.7	7.4±1.5	21.4±1.2*/ ^●●●^
		Spleen	16.6±0.7	15.5±1.3	14.7±1.8	12.3±0.9	12.4±1.6
CD4^+^		Tumour	4.5±0.2	5.6±0.5	6.4±1.0	6.1±0.4	5.2±0.7
		Spleen	13.4±2.3	14.6±1.7	13.7±1.5	14.5±2.1	15.3±1.5
CD8^+^		Tumour	4.9±0.4	5.7±0.4	5.7±0.8	7.6±0.5	13.8±1.1***/ ^●●●^
		Spleen	4.0±1.0	6.1±0.1	5.2±0.6	7.4±0.6	10.1±0.8**/ ^●^
							
CD19^+^	B16F10	Tumour	12.5±2	8.8±2.4	20.3±1.9	26.1±3.1	54.1±2***/ ^●●●^
		Spleen	46.6±3.1	46.7±2.3	51.9±2.9	53.3±1.6	68.7±1.4***/ ^●●^
DX5^+^/CD3^+^		Tumour	6.1±0.9	6.7±0.9	4.4±0.2	5.1±1.4	15.1±1.7**/ ^●●^
		Spleen	1.0±0.1	1.2±0.1	1.1±0.1	1.4±0.2	4.7±0.1***/ ^●●●^
DX5^+^/CD3^-^		Tumour	2.4±0.2	7.0±1.5	10.9±1.2	11.5±0.9	9.9±0.4**
		Spleen	2.4±0.6	2.8±0.4	3.3±0.1	4.8±0.1	5.7±0.4**
CD11c^+^		Tumour	6.3±1.6	5.3±1.9	3.2±1.0	10.3±1.5	24.8±1.8***/ ^●●●^
		Spleen	5.0±0.6	4.9±0.6	3.8±0.3	4.8±0.7	9.5±0.5***/ ^●●●^
F4/80^+^		Tumour	4.3±1.4	9.1±0.5	8.1±0.9	10.9±1.4	18.0±2.3***
		Spleen	9.9±0.7	11.5±1.0	8.3±0.6	11.5±0.3	16.0±0.9***/ ^●●●^
CD4^+^		Tumour	4.3±1.0	1.7±0.7	7.7±1.2	7.5±1.5	9.7±1.3
		Spleen	20.9±1.1	22.9±1.6	19.2±0.4	18.5±0.9	18.6±1.1
CD8^+^		Tumour	7.2±1.4	6.6±0.7	9.9±0.5	7.3±2.1	22.5±1.0***/ ^●●●^
		Spleen	13.4±0.7	13.1±0.5	10.8±0.2	11.1±0.6	23.3±0.5***/ ^●●●^

Cells were isolated from CT26 and B16F10 tumours and spleens from treated and untreated control Balb/C and C57BL/6J mice, respectively. They were analysed by flow cytometry, in which 20 000 events were recorded. Data represent the mean percentage of CD19^+^- (B cells), DX5^+^/CD3^+^- (NKT cells), DX5^+^/CD3^−^- (NK cells), CD11c^+^- (DC cells), F4/80^+^- (macrophage cells), CD4^+^- and CD8^+^- (T cells) positive cells at the time of analysis (48 h) post-treatment. Data represent the mean percentage from fourmice. The asterisks (*) indicate significant values of *P <0.05, **P<0.01 and ***P<0.001 as determined by one-way ANOVA following Bonferroni's multiple comparison of pEEVGmCSF-b7.1 compared with untreated tumour. The asterisks (●) indicate significance values of ^●^*P*<0.05, ^●●^*P*<0.01 and ^●●●^*P*<0.001 as determined by one-way ANOVA following Bonferroni's multiple comparison of pEEVGmCSF-b7.1 compared with the standard vector pGT141GmCSF-b7.1. Similar results were obtained in two independent experiments.

Abbreviations: ANOVA, analysis of variance.
