# Supplementary material for: Non-viral immune electrogene therapy induces potent antitumour responses and has a curative effect in murine colon adenocarcinoma and melanoma cancer models
Source: Gene Ther. 2014 Nov 6;22(1):29–39. doi: 10.1038/gt.2014.95 (PMC4289754; doi:10.1038/gt.2014.95)
Supplement: Supplementary Information [file gt201495x1.docx]

**Supplementary Figures/ Tables**

**Figure S1**

**Plasmid constructs.** Schematic representation of the four circular plasmids used during this study **a.** pEEV: This is the backbone of the plasmid. The EEV plasmid was created by incorporating a Semliki Forest virus DNA replicase sequence (Non structural proteins 1-4), A nuclear localization sequence (NLS) was also incorporated to allow for more efficient nuclear targeting, a 26S subgenomic promoter (26S), the entire SFV capsid gene; which functions as a self-cleaving translation enhancer, capsid (CAP) gene, SV40pA, an Ori colE1 (Ori) and an ampicillin resistance cassette (AmpR). **b.** pEEVGmCSF-b7.1: The EEV plasmid was created by incorporating a Semliki Forest virus DNA replicase sequence (Non structural proteins 1-4), A nuclear localization sequence (NLS) was also incorporated to allow for more efficient nuclear targeting, a 26S subgenomic promoter (26S), the entire SFV capsid gene; which functions as a self-cleaving translation enhancer, capsid (CAP) gene, GmCSF-IRES-b7.1; coding for granulocyte-macrophage colony stimulating factor-internal ribosome entry site- b7.1 co-stimulatory immune molecule, SV40pA, an Ori colE1 (Ori) and an ampicillin resistance cassette (AmpR). **c.** pMG: The pMG plasmid was purchased from InvivoGen. It incorporates the Human Elongation Factor-1α (EF-1α)/Human T-Cell Leukemia Virus Type 1 Long Terminal Repeat (HTLV) hybrid promoter (hEF1CCCa/HTLVp), a multicloning site (MCS), Simian virus 40 late polyadenylation signal (SV40pA). **d.** pGT141GmCSF-b7.1: The pGT141GmCSF-b7.1 was purchased from InvivoGen. It incorporates the HEF1CCCa/HTLVp promoter, GmCSF, SV40, Ori, human CMV-IA promoter, SpAn, Intron A, b7.1, ECM IRES, hygromycin resistance cassette (HygroR). The GmCSF and b7.1 transcriptionally controlled from the HEF1CCCa/HTLVp and CMV-IA promoters respectively.

**Figure S2**: Therapeutic effect on established CT26 at various starting tumour sizes (n=6/group). pEEVGmCSF-b7.1 (a) starting treatment volume approximately 100mm^3^ (n=4 out of 6 mouse survival post 100 days) pEEVGmCSF-b7.1 (b) approximately 150mm^3^ (4 out of 6 survival post 100 days) and pEEVGmCSF-b7.1 (c) approximately 200mm^3^  (3 out of 6 survival post 100 days)

**Table S1** FACS antibody list used in study
