# Supplementary figures and images for: Non-viral immune electrogene therapy induces potent antitumour responses and has a curative effect in murine colon adenocarcinoma and melanoma cancer models
Source: Gene Ther. 2014 Nov 6;22(1):29–39. doi: 10.1038/gt.2014.95 (PMC4289754; doi:10.1038/gt.2014.95)

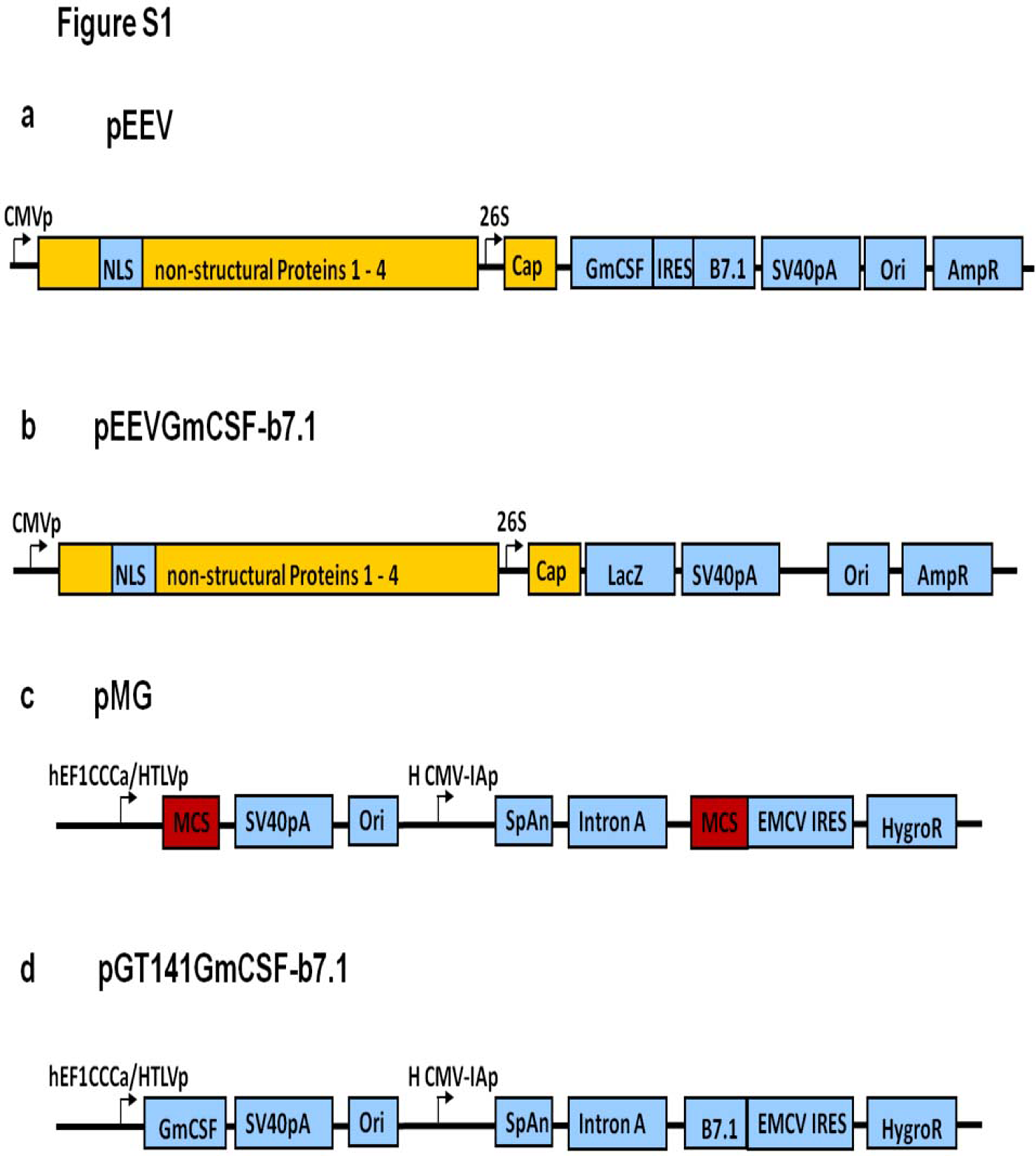

Supplement: Supplementary Figure S1 [file gt201495x2.tif]

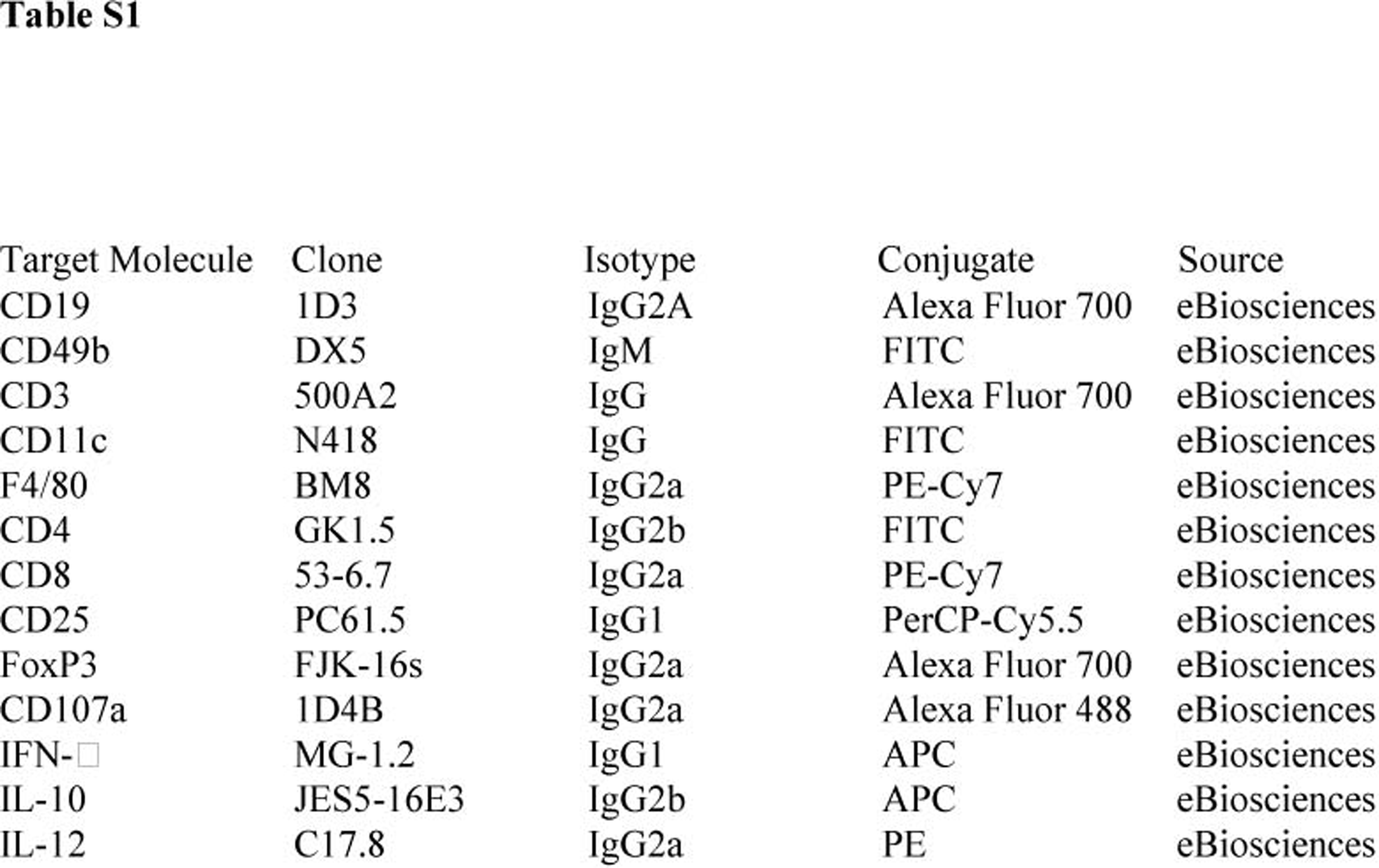

Supplement: Supplementary Table S1 [file gt201495x4.tif]
